# ScFvs as Allosteric Inhibitors of VEGFR-2: Novel Tools to Harness VEGF Signaling

**DOI:** 10.3390/ijms19051334

**Published:** 2018-05-01

**Authors:** Kurt Ballmer-Hofer, Caroline A.C. Hyde, Thomas Schleier, Dragana Avramovic

**Affiliations:** Laboratory of Biomolecular Research, Paul Scherrer Institut, 5232 Villigen, Switzerland; cachyde@gmail.com (C.A.C.H.); thomschleier@gmail.com (T.S.)

**Keywords:** angiogenesis, VEGFR-2, VEGF, scFv, inhibition, receptor downregulation

## Abstract

Vascular Endothelial Growth Factor Receptor 2 (VEGFR-2) is the main mediator of angiogenic signaling in endothelial cells and a primary responder to VEGF. VEGF dependent VEGFR-2 activation regulates endothelial cell migration and proliferation, as well as vessel permeability. VEGF is presented as an antiparallel homodimer, and its binding to VEGFR-2 brings two receptors in close proximity. Downstream signaling is triggered by receptor dimerization, kinase activation, and receptor internalization. Our aim was to further investigate allosteric inhibition using binders targeting extracellular subdomains 4–7 of VEGFR-2 as an alternative to existing anti-angiogenic therapies, which rely on neutralizing VEGF or blocking of the ligand-binding site on the receptor. We applied phage display technology to produce single chain antibody fragments (scFvs) targeting VEGFR-2. Selected antibody fragments were characterized using biophysical and biological assays. We characterized several antibody fragments, which exert their inhibitory effect of VEGFR-2 independent of ligand binding. These reagents led to rapid clearance of VEGFR-2 from the cell surface without kinase activation, followed by an increase in intracellular receptor-positive vesicles, suggesting receptor internalization. Our highly specific VEGFR-2 binders thus represent novel tools for anti-angiogenic therapy and diagnostic applications.

## 1. Introduction

Angiogenesis is the process of creation of new blood capillaries from pre-existing vasculature. It occurs throughout the life of higher organisms, in both healthy and pathological conditions. 

Metabolically active tissues require blood vessels in close proximity for exchange of nutrient and metabolites. Owing to the fact that aberrant angiogenesis is implicated in a number of pathologies, research interest in its therapeutic control has steadily increased over the past decades. Approaches which deal with insufficient angiogenesis carry curative potential for ischemic heart disease, peripheral arterial disease, and wound healing, whereas excessive angiogenesis can be targeted in pathologies such as cancer, certain ophthalmic conditions, and rheumatoid arthritis. 

Vascular endothelial growth factors (VEGFs) and their receptors (VEGFRs) are required for vascular and lymphatic homeostasis, but their aberrant signaling can give rise to pathological angiogenesis. Different members of the VEGF protein family bind VEGFR-1, -2, and -3, respectively, thereby regulating endothelial cell survival, proliferation, differentiation, and migration. VEGFR-2 is the major mediator of angiogenic signaling in endothelial cells, hence various tactics to target VEGF/VEGFR-2 signaling have been developed. However, most currently used strategies show significant side effects. Inhibition of VEGF signaling by ligand sequestering agents (bevacizumab) [1] or VEGFR-2 blocking antibodies (ramucirumab) [2,3] can be bypassed by high ligand concentration. On the other hand, classical low molecular weight tyrosine kinase inhibitors, such as sunitinib, widely used to suppress VEGFR-2 activity, are not exclusively specific for VEGFR-2 [4]. 

The extracellular domain (ECD) of VEGF receptors comprises seven immunoglobulin homology domains (Ig-domains). Previous studies have shown that domains D2 and D3 of the ECD are required for ligand binding [5,6] while D4–7 are required for receptor dimer stabilization and the orientation of receptor monomers in active dimers [7,8]. The significance of such homotypic interactions in the receptor ECD, for VEGFR-2 activation, has been previously demonstrated and reported by our group [7,8,9,10]. Consequently, targeting of allosteric regulatory sites in D4–7 of VEGFR-2 by mutation or using inhibitory molecules, such as DARPins [11,12] and antibodies [13], have demonstrated successful inhibition of VEGF signaling. 

Phage-displayed scFvs offer several advantages compared to full-size monoclonal antibodies, including inexpensive production in *E. coli*, simple genetic modification [14], and reduced immunogenicity. The smaller size of scFv fragments compared to full-size immunoglobulins provides fast target delivery and even penetration into target tissues, e.g., into tumors [15,16]. Since scFvs are more readily cleared from circulation, their exposure to healthy tissue is vastly reduced [17,18], making their application particularly attractive for imaging [19], drug delivery [20,21], and radionuclide- or drug-coupled nanobody delivery. Furthermore, scFv selection and production can be standardized, and thereby lends itself to automated and high throughput approaches [14].

The work presented in this study is based on our past functional characterization of VEGFR-2 activation. Our new reagents specifically target D4–7 of the ECD. We describe the production and characterization of scFv antibody fragments, and their effect on receptor kinase activity and receptor trafficking. In addition, we studied the biological output of VEGFR-2 following scFv administration in vitro.

## 2. Results

### 2.1. Selection, Production, and Purification of ScFv Antibodies

The synthetic ETH-2 Gold library was used for three rounds of selection of scFvs against hVEGFR-2. A total of 288 single clones were tested with ELISA, and 40 clones recognizing domains of interest were sequenced. ScFvs bearing unique sequences were further characterized. Three scFvs targeting different extracellular subdomains were chosen for further analysis (Table 1). ScFvs G3 and F1 were generated with the ETH-2 Gold phage library [22] using human VEGFR-2 ECD as antigen. ScFv A7 [23], previously selected and characterized by Böldicke et al., was selected with an antigen-biased immune V-gene phage display library which originated from murine lymphocytes, and was subcloned into pDN332 vector. As scFvs G3 and F1 were selected from an entirely different library than scFv A7, the sequences also differ. *E. coli* strain Mach-1 was used for large scale production, where soluble scFvs were purified utilizing an Äkta system with metal (Ni; scFv A7) or protein affinity chromatography (protein A; scFv G3 and scFv F1).

### 2.2. Binding of ScFvs to Recombinant and Endogenous VEGFR-2

**Figure 1:**
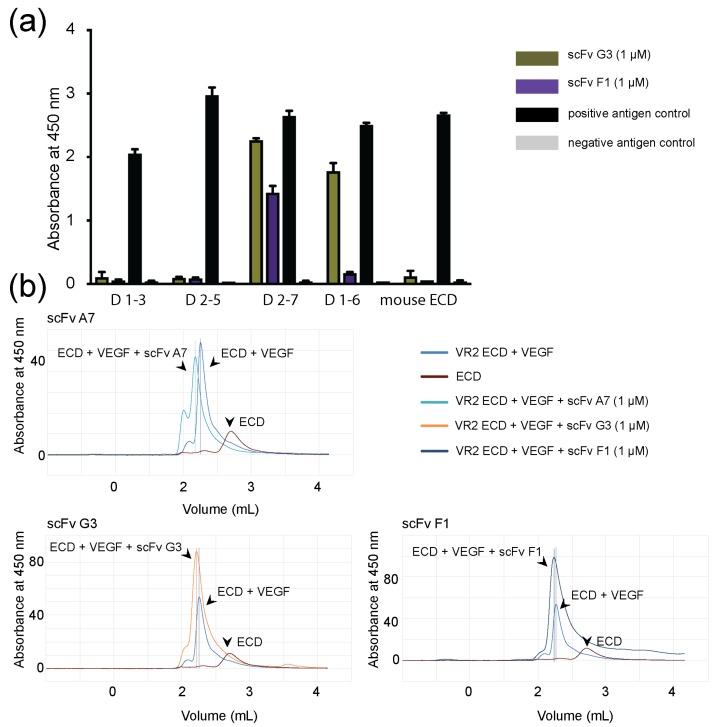
Analysis of scFv binding to the VEGFR-2 ECD (**a**) ELISA results show VEGFR-2 domain specificity, shown as absorbance at 450 nm. ELISA plates were coated with different length VEGFR-2 ECD constructs and exposed to 1 μM of scFvs. Absorbance signal represents specific binding of scFvs to VEGFR-2 ECD construct. Antigen controls demonstrate wells with and without VEGFR-2 ECD construct. Presented results are the mean of three independent experiments where error bars represent ± standard deviation (SD); (**b**) Fluorescence size-exclusion chromatography results show ScFvs binding to VEGFR-2 ECD/VEGF complex. ScFvs (1 µM) were incubated with VEGFR-2 ECD/VEGF complex, and the mixture was analyzed with FSEC. VEGFR-2 ECD/VEGF complex and VEGFR-2 ECD were used as controls. The incubation with scFvs led to the shift in the peak position to the left, demonstrating the formation of new larger species in combination with VEGFR-2 ECD/VEGF and the bound scFvs. ScFv addition did not lead to a shift in the peak position to the right, indicating that binding of scFvs did not disrupt the complex.

**Figure 2:**
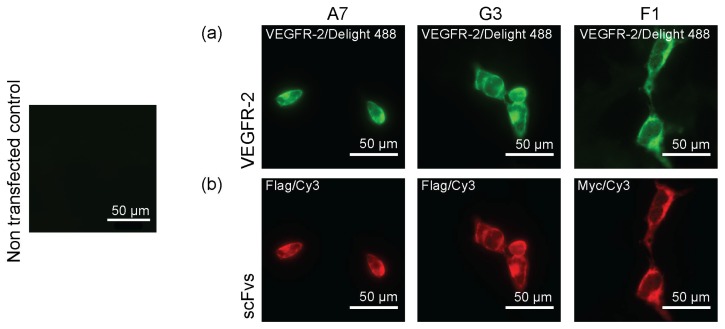
ScFvs bind VEGFR-2 expressed on the surface of HEK293 cells. (**a**) VEGFR-2 was detected with commercial primary VEGFR-2 antibody and fluorescently-labeled secondary antibody (green); (**b**) For ScFvs staining, myc-tag (scFvs G3 and F1) and flag-tag (scFv A7) primary antibodies were used, followed by secondary fluorescently-labeled antibodies (red). Bound ScFvs show the same binding pattern as commercial VEGFR-2 antibody. Non-transfected HEK293 cells were used as negative control.

Binder specificity was investigated with ELISA using different length constructs of human VEGFR-2 (hVEGFR-2) ECD, whereas mouse VEGFR-2 (mVEGFR-2) ECD served as negative control (Figure 1a). Selected antibody fragments G3 and F1 were specific for human D6 and D7, respectively, but were not binding mVEGFR-2 ECD. The binding specificity for scFv A7 was defined by size-exclusion chromatography (SEC) (Figure S1) where scFv A7 was incubated with different length constructs of VEGFR-2 ECD and purified on a Superdex S-200. Collected peak fractions were concentrated and resolved on 12% SDS-PAGE gels. ScFv A7 recognized all constructs containing D2–3. Fluorescence size-exclusion chromatography (FSEC) (Figure 1b) shows a shift of the peak to the left, demonstrating the formation of larger protein complexes. This indicates that scFvs bound, but did not disrupt, the VEGFR-2 ECD/VEGF complex, illustrating that scFvs and VEGF did not compete for the binding site. Binding to live cells was observed in transiently VEGFR-2 transfected HEK293 cells by immunostaining. Here, we used commercially available antibodies to label VEGFR-2 and to visualize scFvs binding to the receptor on the cell surface. The green signal represented VEGFR-2 immunostaining with primary VEGFR-2-specific antibody and secondary fluorescently-labeled antibody. The red signal represented binding of myc- and flag-tag antibodies to scFvs G3 and F1, and scFv A7, respectively, accompanied with the fluorescently-labeled secondary antibody. Non-transfected HEK293 cells were used as negative control. Altogether, this data confirmed that scFvs recognize VEGFR-2 on the surface of the cells (Figure 2). Affinities of the scFvs were measured by isothermal titration calorimetry (ITC) (Figure S2). The advantage of using ITC compared to surface plasmon resonance (SPR) is that both binding players are in solution, and that the binding affinities are measured directly during binding. The calculated *K*d values are summarized in Table 1.

### 2.3. Functional Inhibition of VEGFR-2 Phosphorylation with ScFvs

**Figure 3:**
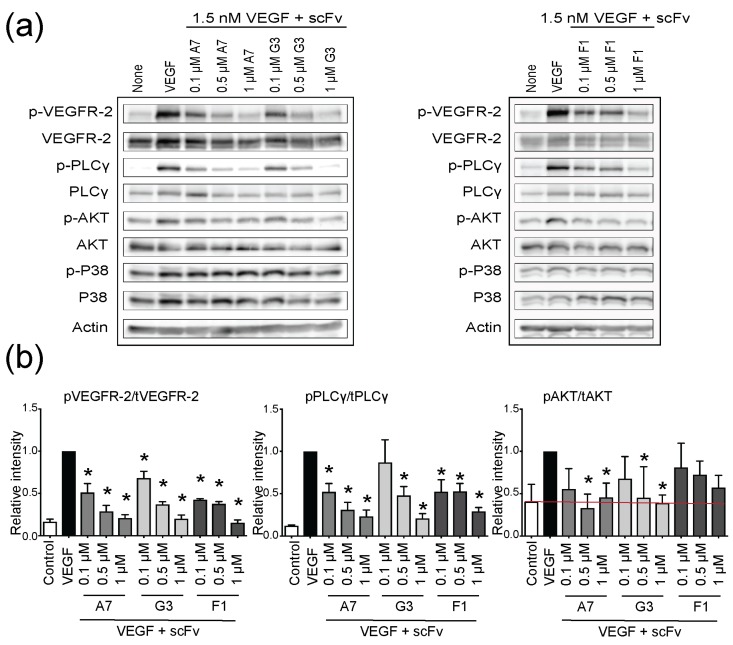
ScFvs inhibit VEGF-induced signaling in PAE-KDR cells. (**a**) Western blot results show the effect of pretreatment with increasing concentrations of scFvs and demonstrate inhibition of phosphorylation of VEGFR-2, PLCγ, and AKT in a dose-dependent manner, without affecting total protein levels; (**b**) Data was quantified with ImageJ and further analyzed with GraphPad Prism 7. Presented results are the mean of three independent experiments where error bars represent ± standard deviation (SD). The statistical significance was investigated with ordinary 1-way ANOVA using Dunnett’s test, and indicated by * representing *p* < 0.05. The red line references non-stimulated control.

We used kinase assays to functionally characterize selected antibody fragments. PAE-KDR cells that recombinantly express VEGFR-2 were exposed to increasing concentrations of scFvs in the presence of physiological concentrations of VEGF. Incubation with VEGF alone led to rapid VEGFR-2 phosphorylation (Figure 3), and that effect was inhibited by pretreatment with scFvs. Upon addition of scFv, VEGFR-2 phosphorylation was downregulated in a dose-dependent manner, without affecting overall VEGFR-2 expression. As signal output of VEGFR-2, we examined expression and phosphorylation of PLCγ, AKT, and p38 mitogen-activated protein kinase (MAPK). While PLCγ phosphorylation was clearly inhibited, the level of AKT phosphorylation was only marginally affected by scFvs. VEGF-mediated activation of p38 did not show a statistically significant change upon scFv addition. Taken together, treatment of PAE-KDR cells with scFv antibody fragments suppressed VEGF-dependent receptor phosphorylation and downstream signaling in a dose-dependent manner without showing an effect on total protein levels (Figure 3a).

### 2.4. Effect of ScFvs on In Vitro Angiogenesis

**Figure 4:**
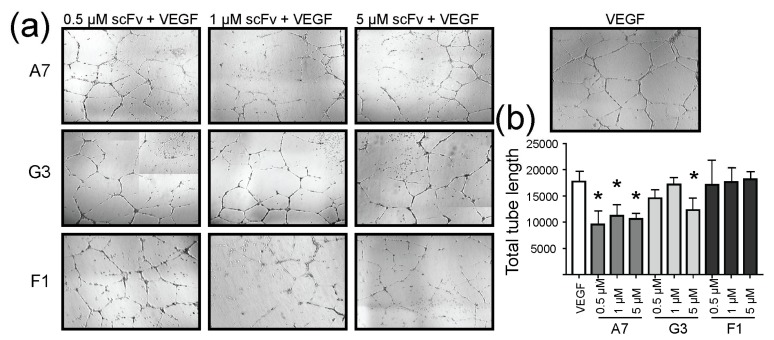
ScFvs inhibit VEGF-induced development of tube-like structures in HUVECs. (**a**) Microscopy images at 4× magnification show tubular structures formed in the presence of VEGF. Tube formation was disrupted in the presence of increasing concentrations of scFvs; (**b**) Image analysis quantifies the length of ligand-induced tubes in the presence and absence of scFv antibody fragments. Presented results are mean of three independent experiments, where error bars represent ± standard deviation (SD). The statistical significance was investigated with ordinary 1-way ANOVA using Dunnett’s test and indicated by * representing *p* < 0.05.

We examined the effect of our antibody fragments on the formation of capillary-like structures formed by endothelial cells exposed to VEGF. HUVECs were embedded in Matrigel incubated for 18 h in the presence or absence of scFvs. HUVECs incubated with VEGF-containing Matrigel led to the formation of tube-like structures, while treatment with increasing concentration of scFvs significantly inhibited this effect (Figure 4a). Endothelial cell migration in response to VEGF represents a critical step in the formation of new blood vessels. In wounded HUVEC monolayers, VEGF-induced migration was decreased upon treatment with scFvs (Figure S3).

### 2.5. VEGF and ScFvs Promote Internalization of VEGFR-2

**Figure 5:**
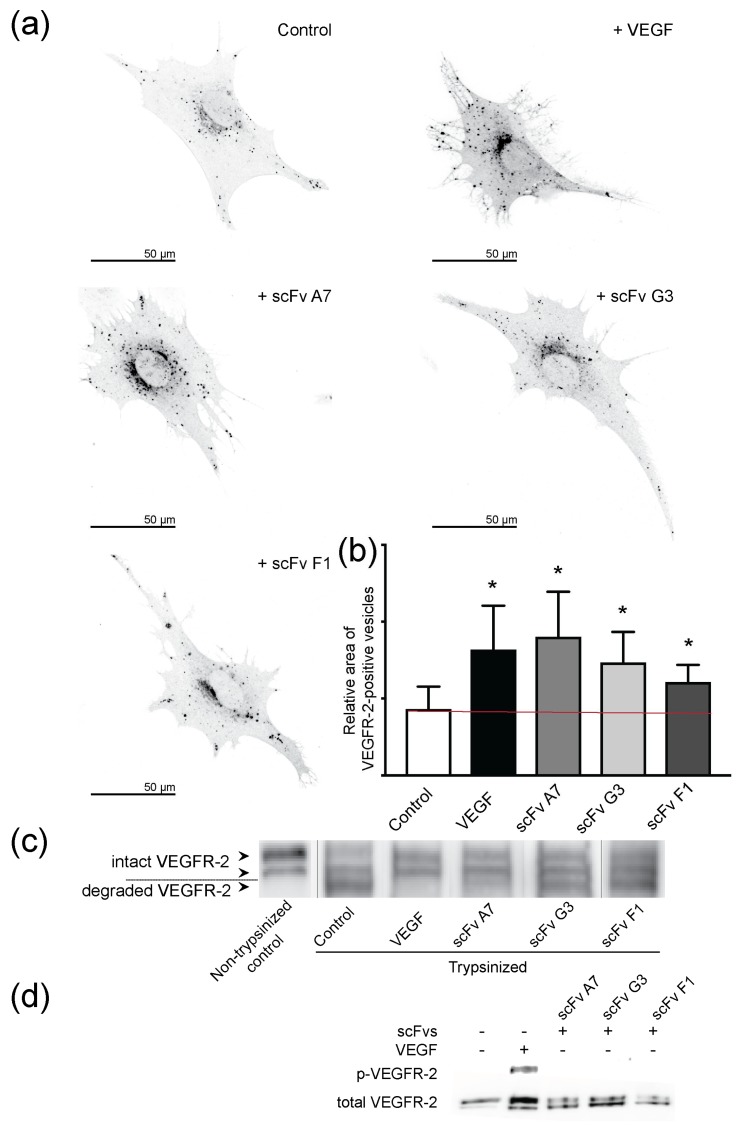
VEGF and scFvs promote VEGFR-2 internalization. (**a**) Immunostaining of VEGFR-2 expressing PAE-KDR cells show receptor internalization following VEGF or scFv administration. PAE-KDR cells were fixed and stained for 45 min after addition of VEGF or scFvs; (**b**) Area of VEGFR-2 positive vesicles relative to total cell area was analyzed by Squassh. Statistical data analysis was performed using GraphPad Prism 7. Data show representative images of 25 independent samples. Error bars represent ± SD. The statistical significance based on a Student’s *t*-test is indicated by * representing *p* < 0.05. The red line references non-stimulated control; (**c**) Western blot analysis of control and trypsin-treated cells using VEGFR-2-specific antibody. Cells were treated with VEGF or scFvs for 45 min. Top two arrows on the left of the blot indicate bands representing intact receptor, bottom arrow points to band of degraded receptor. (**d**) Kinase assay. SDS-PAGE gel shows kinase activity of VEGFR-2 determined with phospho-tyrosine-specific antibody in PAE-KDR cells. As controls we showed non-stimulated and VEGF-stimulated kinase activation. Bottom row shows total level of VEGFR-2 for the same gel.

Based on our recently published work on DARPin^®^ VEGFR-2-domain D4b-induced receptor internalization [12], we studied scFv-induced internalization of VEGFR-2 in the presence and absence of VEGF. We used the same two approaches to determine receptor internalization as published [12]. First, we determined receptor uptake into intracellular vesicles in PAE-KDR cells and measured VEGFR-2 positive vesicle area (Figure 5). A significant increase of VEGFR-2 positive vesicle area upon addition of either VEGF or scFvs was observed.

To confirm that receptor internalization correlates with VEGFR-2 removal from the cell surface upon VEGF or scFv binding, we trypsinized membrane-bound receptor on intact cells. Membrane-bound extracellular VEGFR-2 was digested, while internalized protein was protected and remained intact. In untreated control cells, only full-length VEGFR-2 was observed (Figure 5c, lane 1), while trypsin treatment led to the degradation of the receptor in control cells, as indicated by the two lower bands (lane 2) of the SDS-PAGE gels. By contrast, VEGF or scFv A7 incubation led to VEGFR-2 protection from trypsin degradation, and predominantly the bands corresponding to intact VEGFR-2 were observed (lanes 3 and 4). ScFvs G3 and F1 induced partial VEGFR-2 internalization, and bands indicating protected and degraded receptor were present (lanes 5 and 6). These data agree with Squassh analysis of VEGFR-2 internalization (Figure 5a) showing a stronger internalization effect was induced with scFv A7, compared to scFvs G3 and F1. Furthermore, scFv A7 displayed a lower dissociation constant compared to scFvs G3 and F1, which mirrors its increased inhibitory effect observed at the functional level (Table 1). Moreover, we showed that scFv incubation, on its own, had no impact on VEGFR-2 activation (Figure 5d).

In order to exclude that toxic side-effects were responsible for inactivation of VEGFR-2 signaling, cell viability assays were performed. All scFvs proved non-toxic at working concentrations of 0–10 μM, with a cell viability >90% (data not shown).

So far, we showed the selection, production, and purification process, accompanied with biophysical and biological characterization of scFvs targeting VEGFR-2. The inhibitory effect of described antibody fragments was tested in kinase and angiogenesis assays. Moreover, we demonstrated that incubation with scFvs leads to ligand independent VEGFR-2 internalization.

## 3. Discussion

The VEGF/VEGFR-2 signaling axis represents an essential and attractive target to manipulate pathological angiogenesis in vivo. Ramucirumab is a monoclonal antibody developed from a human Fab antibody fragment targeting VEGFR-2 [24,25], and approved for the treatment of advanced gastric cancer, gastroesophageal junction adenocarcinoma cancer, non-small cell lung cancer, and metastatic colorectal cancer [2,3,26,27]. Furthermore, promising studies with antibodies and DARPins targeting VEGFR-2 have been previously published [11,13]. We set out to generate novel VEGF/VEGFR-2 inhibitors based on VEGFR-2 specific antibody fragments that allosterically block receptor activation.

Described antibody fragments specifically recognize different subdomains of the VEGFR-2 ECD. ScFvs A7, G3, and F1 exclusively target D2–3, D6, and D7, respectively. Selected scFvs showed species-specificity by recognizing both purified recombinant human receptor ECD protein and hVEGFR-2 transiently expressed on the surface of HEK293 cells. Antibody fragment binding did not disrupt VEGFR-2 ECD/VEGF complex formation, but rather gave rise to larger protein complexes, demonstrating that scFvs and VEGF bind to different sites on the receptor ECD. The best binders demonstrated affinities in the nanomolar range, comparable to those of previously published scFvs [28,29]. In order to use the described antibody fragments for further in vivo testing, additional optimizations, including affinity maturation, may be required. Alongside binding affinity, scFv stability may represent a further challenge. Various strategies were developed to address this issue, including the introduction of extra disulfide bonds [30,31], and modification of the peptide linker in both length and composition [32,33].

Nonetheless, we showed that the presently investigated ScFvs significantly suppressed VEGF-induced VEGFR-2 phosphorylation and downstream signaling. Selected antibody fragments also showed consistent inhibition of biological receptor output, such as inhibition of cell migration and tube formation of HUVECs, indicative of specific inhibition of VEGF-driven angiogenesis in vitro.

Most interestingly, internalization studies revealed a possible cellular mechanism of scFv-mediated VEGFR-2 inhibition. Recently, our group published results showing that DARPin^®^ domain D4b induces VEGFR-2 internalization independently of VEGF [12]. Here, we show the same effect with scFvs targeting D2–3, D6, or D7 (Figure 5). Antibody fragments binding to VEGFR-2 led to receptor internalization, as demonstrated by an increase in the relative area of intracellular VEGFR-2-positive vesicles (Figure 5a), and the clearance of the receptor from the cell surface (Figure 5c) without an impact on VEGFR-2 activation (Figure 5d). Internalization independent of ligand binding and thus, of kinase-inactive receptor, represents a novel property of the scFvs described here. This finding indicates that internalization is not binder or domain specific, but is a shared property among all allosteric VEGFR-2 inhibitors studied in the course of our studies. The mechanism of receptor downregulation is shared with other receptor tyrosine kinase antibodies specific for HER2 or EGFR [34,35]. Drebin et al. [36] and Hudziak et al. [37] have shown that HER2-specific antibodies reduced the amount of receptor expressed on the surface of cancer cells. Similar to antibodies specific for HER2 [38], the allosteric effect of our scFvs might provoke structural changes in the receptor ECD resulting in receptor internalization and clearing from the plasma membrane. Previously, receptor clustering followed by unspecific internalization was also described for another HER2-specific antibody, pertuzumab [39]. We showed previously in live cells that VEGFR-2 forms dimers, also in the absence of ligand [40]. We assume, therefore, that receptor crosslinking is a possible mechanism underlying VEGFR-2 internalization.

Similarly to earlier described Ankyrin repeat-derived binding scaffolds [11,12], scFvs, due to their high specificity and selectivity, represent excellent tools for specifically targeting the extracellular domain of membrane-bound molecules. In addition, scFvs can be easily reformatted to Fab or full-size immunoglobulins to modulate bioavailability in a host organism. Due to the demonstrated receptor internalization property, such antibody fragments might also be useful as immunotoxin or nanoparticle-antibody conjugates to deliver therapeutic cargo to diseased cells. There are several scFv-based strategies undergoing preclinical and clinical trials at present [41,42,43,44], proving that such an approach reaches beyond basic research and is applicable for novel clinical applications.

## 4. Materials and Methods

### 4.1. Cell Culture

Porcine aortic endothelial cells overexpressing VEGFR-2 (PAE-KDR) and human embryonic kidney epithelial 293 cells (HEK293) were cultured as monolayers in Dulbecco’s modified Eagle’s medium (DMEM; BioConcept, Basel, Switzerland) enriched with 10% fetal bovine serum (FBS) and 1% penicillin streptomycin. Human umbilical vein endothelial cells (HUVECs; Lonza, Walkersville, MD, USA) were cultured in EGM-2 medium (Lonza). Cells were grown in humidified incubator at 37 °C and exposed to 5% CO_2_.

### 4.2. Transient Transfection

HEK293 cells at 60% confluency were transiently transfected with pBE plasmid bearing VEGFR-2 sequence with FuGENE (Promega, Madison, WI, USA) in Opti-MEM medium (Life Technologies, Carlsbad, CA, USA) in a 2:3 ratio of DNA/FuGENE. Immunostaining was performed 24 h after transfection. Antibodies used were as follows: tVEGFR-2 (ab11939, Abcam, Cambridge, UK), myc-tag (2276S, Cell Signaling, Danvers, MA, USA), flag-tag (F3165, Sigma-Aldrich, St. Louis, MA, USA). Fluorescently-labeled Dylight 488 and Cy3 were acquired from Abcam.

### 4.3. VEGFR-2 Kinase Activity Assay

Serum starved PAE-KDR cells were incubated for 10 min with 1.5 nM VEGF (in all experiments VEGF-A_165_ was used) with or without 30 min pretreatment with scFvs. Cells were lysed with lysis buffer (50 mM Tris (pH 7.5), 100 mM NaCl, 0.5% (*w*/*v*) Triton X-100) supplemented with protease inhibitor cocktail (Complete Mini EDTA-free, Roche) and phosphatase inhibitors (200 μM Na_3_VO_4_, 20 μM phenylarsine oxide). Lysates were diluted with 5× loading buffer (0.25 M Tris-HCl pH 6.8, 0.5 M DTT, 10% SDS, 50% glycerol, 0.5% bromophenol blue). Samples were boiled at 50 °C for 30 min, and resolved by 7% SDS PAGE, transferred to PVDF membranes (GE Healthcare, Piscataway, NJ, USA), and immunodecorated with primary antibodies (dilution 1:1000), followed by secondary alkaline phosphatase-coupled antibodies (1:10000). Immunoblots were developed with Novex AP Chemiluminescent Substrate (Invitrogen, Carlsbad, CA, USA). Amersham Imager 600 (GE) was used for analysis. Antibodies used were as follows: pVEGFR-2 (2478, Cell Signaling), tVEGFR-2 (2479, Cell Signaling), pPLCγ1 (2821, Cell Signaling), tPLCγ1 (2822, Cell Signaling), pAKT (4060, Cell Signaling), tAKT (4051, Cell Signaling), p38 (4631, Cell Signaling), and tp38 (9212, Cell Signaling). Protein marker used was PageRuler™ Plus Prestained Protein Ladder, 10 to 250 kDa (26619, ThermoFisher, Waltham, MA, USA).

### 4.4. Immunofluorescence Microscopy

Cells were grown to 60% confluency on glass coverslips coated with poly-l-lysine (P4707, Sigma-Aldrich). Cell fixation at 37 °C for 20 min was performed with 3.7% formaldehyde in phosphate-buffered saline (PBS), followed by 10 min permeabilization with 0.1% NP-40 in PBS, and 20 min blocking in 5% BSA/PBS at room temperature. For immunostaining, primary and fluorescently-labeled secondary antibodies were diluted in blocking solution, and samples were embedded. Images were acquired with an Olympus IX81 equipped with an Andor iXonEM camera (Figure 2), and with Leica SP5 laser scanning confocal microscope (Figure 5).

### 4.5. Phage Display and Selection of ScFv Antibodies

The synthetic ETH-2 Gold library was kindly provided by Prof. Dario Neri from ETH, Switzerland.

Phage display using the ETH-2 Gold phage library was performed as previously described [22,45]. In short, immunotubes (470319K, Nunc, Wiesbaden, Germany) were coated with extracellular domain of VEGFR-2 (made in-house) at a concentration of 10^−6^ M. Antigen-exposing tubes were incubated with the phage for 1 h at room temperature, followed by washing steps, performed to remove unbound and weakly bound phages. Specific binders were eluted, amplified in *E. coli* Tg1, and used for next two rounds of selection. After the third round, bacteria were diluted and plated to obtain single clones used for scFvs production from *E. coli* supernatants. ELISA was used to screen for binders to VEGFR-2 ECD, as described below. Positive binders with unique sequences were identified and used to infect the *E. coli* non-suppressor strain Mach1 for the large-scale production. ScFv A7, used in this study, was obtained from an antigen-biased immune V-gene phage display library generated from murine lymphocytes [23], and subcloned in pCANTAB 5E, as described below.

### 4.6. Subcloning of pCANTAB 5E ScFv A7

The obtained scFv A7 was in the plasmid pCANTAB 5E [23]. To subclone it into the pDN332 vector, two-step PCR cloning was performed. Amplification primers were pCpD-for 5′-GTT ATT ACT CGC GGC CCA GCC GGC CAT GGC CCA GGT GAA ACT GC AGG AGT C-3′ and pCpD-rev 5′-TCC CCC CTG GTT CGA CCT CGA CTG GCA GGA TCC GCG CCG GCG TCT A-3′. NotI/SfiI (New England Biolabs) double digestion was carried, followed by ligation and transfection of *E. coli* Mach1 cells.

### 4.7. Expression and Purification of ScFvs

Soluble scFvs were expressed in *E. coli* Mach1 cultured at 37 °C in 2×YT medium supplemented with 0.1% glucose and 100 μg/mL ampicillin. When bacterial growth reached 0.8 OD600, the expression was induced with 1 mM IPTG. *E. coli* was further propagated at 30 °C for 10 h, followed by centrifugation at 4000 *g*. A fully-automated liquid chromatography instrument Äkta (GE Healthcare) was used for affinity chromatography purification. ScFv-enriched supernatant was filtered and purified with immobilized metal affinity chromatography (IMAC) using protein A (GE) or with Ni-affinity chromatography (GE). Buffers used were as follows. For protein A chromatography purification: binding buffer, 20 mM Tris pH 8.0; washing buffer, 20 mM glycine pH 6.0; and elution buffer, 0.1 M glycine pH 2.5. For Ni-affinity chromatography purification: binding buffer, 50 mM sodium phosphate, 300 mM NaCl, 10 mM imidazole, pH 7.4; and elution buffer, 50 mM sodium phosphate, 300 mM NaCl, 500 mM imidazole, pH 7.4. Eluted fractions (2 mL) containing scFvs were pooled together, concentrated up to 1 mg/mL, and dialyzed overnight against PBS, pH 7.4, at 4 °C. NanoDrop A280 measurements were used for estimating soluble protein concentrations, and the obtained yield of scFvs was in the range of 1–4 mg from 1 L of bacterial culture.

### 4.8. Enzyme-Linked Immunosorbent Assay (ELISA)

Recombinant VEGFR-2 ECD proteins D1–3, D2–5, D2–7, and D1–6 were used in ELISA to determine binders’ specificity for specific VEGFR-2 ECD subdomains. Maxisorp plates (Nunc) were coated overnight with 3 g/mL of the antigen. Extensive washing steps performed to avoid false positive signal were followed by blocking in PBS/1% BSA/0.1% Tween 2 and exposure to 1µM scFvs for 2 h at room temperature. As scFvs G3 and F1 were caring myc-tag, myc-tag antibody (2276S, Cell Signaling) as primary and anti-mouse IgG HRP-linked (7074S, Cell Signaling) as secondary antibodies were used for binding detection. All used antigens were His-tagged, hence, we used His-tag (34,660, Qiagen, Hilden, Germany) as primary antibody and anti-mouse IgG HRP-linked as secondary antibody to detect antigen immobilization on the plate surface. As negative control, we used wells without antigen. Reaction was induced by peroxidase substrate (ThermoFisher) and quenched by 2 M sulfuric acid (H_2_SO_4_). An automated plate reader (TECAN Safire2) was used to measure OD450 value.

### 4.9. Size-Exclusion Chromatography (SEC)

SEC was performed with a Superdex S-200 size exclusion column (Amersham Pharmacia Biotech, Dübendorf, Switzerland). Samples (500 μL) containing scFv A7 and VEGFR-2 ECD variants at a molar ratio of 2:1 were incubated at 4 °C and injected onto the column. HEPES buffer was used for protein separation, and protein absorption was detected at 280 nm.

### 4.10. Fluorescence Size-Exclusion Chromatography (FSEC)

Ultracentrifuged samples in PBS were applied to a Shodex semi-micro KW404-4F (4.6 Å~300 mm) column, pre-equilibrated with PBS, pH 7.4 at 4 °C. The fractions from the SEC column were passed through a fluorometer (FLD1: excitation/emission 435/470 nm, gain 12; FLD2: excitation/emission 515/535 nm, detector gain 10). Data was analyzed with OriginLab (Origin 9.1).

### 4.11. Binding Affinity Determination by Isothermal Titration Calorimetry (ITC)

The binding enthalpy of scFv proteins was assessed by ITC carried out on a iTC200 calorimeter (MicroCal^®^, Northampton, UK). Experiments were performed at 15 °C. Prior to use, all protein samples were dialyzed extensively against working solution (PBS) and degassed for 10 min. VEGFR-2 ECD protein (15 μM) was used as a titrant in the cell, and the scFvs (400 μM) were used as titrants in the syringe at a tenfold higher concentration. Data was processed with the OriginTM 8.0 software (Microcal Inc., Northampton, MA, USA), using non-linear fitting models to calculate the reaction stoichiometry (N), binding constant (K), enthalpy (DrH), and entropy (DrS).

### 4.12. HUVEC Tube Formation Assay

For tube formation assays, commercially available 15-well μ-Slides (Ibidi, Munich, Germany) were precoated with Geltrex^®^ Matrix (ThermoFisher). After the matrix polymerized, 6000 HUVECs were plated on the plates and maintained in previously described conditions. Increasing concentrations (0.5, 1, and 5 µM) of scFvs were used to test the inhibiting potential. After 18 h, the plates were fixed with 3.7% formaldehyde (FA). Previously described Olympus IX81 microscope was used for obtaining images of tubes-like structures. Images were analyzed using automated image analysis software (http://ibidi.wimasis.com/).

### 4.13. HUVEC Migration Assay

For migration assays, Culture-Inserts from Ibidi, Germany, were used. 21,000 HUVECs were seeded in each chamber and maintained in previously described conditions. The following day, culture inserts were removed, and cells were stimulated with VEGF in the presence and absence of increasing concentrations of scFvs (0.5, 1, and 5 µM). The previously described Olympus IX81 microscope was used for obtaining images of migrating cells. 

### 4.14. Squassh Analysis of VEGFR-2 Internalization

We used Squassh [46] to segment intracellular vesicles of 25 cells per condition, as previously described [12]. Squassh represents an ImageJ plugin that combines segmentation and deconvolution of images in a single step, yielding better results for small objects close to the diffraction limit of the microscope. Squassh Analyst was used for data analysis and normalization of the VEGFR-2 positive vesicle area relative to the total cell area. Prior to Squassh analysis, cells were treated either with 1.5 nM VEGF or 1 μM scFvs for 45 min in starvation media. Used antibodies were VEGFR-2-specific antibody (2479, Cell Signaling) as primary, and fluorescently-labeled Dylight 488 (Abcam) as secondary antibody.

### 4.15. Trypsin Digestion of Cell Surface-Exposed Receptors

PAE-KDR cells were cultured in standard previously described conditions prior starvation and 45 min treatment with 1.5 nM VEGF or 1 μM scFvs. Cells were consequently washed three times with ice-cold PBS, and then incubated for 30 min at 4 °C with freshly prepared trypsin (1 mg/mL, Sigma). The enzymatic reaction was quenched by the addition of soybean trypsin inhibitor (50 mg/mL, Sigma). Cells were scraped off the plate and centrifuged at 500 rpm at 4 °C for 5 min. The cell pellet was lysed in gel electrophoresis sample buffer, heated to 95 °C for 5 min, and VEGFR-2 protein was analyzed by SDS–PAGE and Western blot using VEGFR-2 antibody (2479, Cell Signaling).

### 4.16. Statistical Analysis

Statistical analysis was performed using GraphPad Prism 7 (GraphPad Software Inc., San Diego, CA, USA). Data was investigated by ordinary 1-way ANOVA using Dunnett’s test for multiple comparison and presented as mean ± SD shown by error bars. All results represent mean of three experiments unless indicated differently. Results with *p* values < 0.05 were considered statistically significant and marked with an asterisk (*).

## 5. Conclusions

In summary, a range of novel antibody fragments in the form of highly specific anti-VEGFR-2 scFvs has been described, and the functional effects of allosteric inhibition of VEGFR-2 were characterized. The ability of scFvs to selectively block VEGF signaling via binding to an extracellular allosteric site in the cognate receptor, and their use in antibody-mediated drug delivery systems, document their potential for future therapeutic applications. At the mechanistic level, our findings provide the proof-of-principle that anti-VEGFR-2 agents targeting the membrane-proximal Ig-domains D2–3, D6, or D7, downregulate receptor activity independent of ligand binding and thus represent a novel strategy to target VEGFR-2 signaling.

## Figures and Tables

**Table 1:** Summary table showing CDR3 sequences of heavy and light chains, ETH-2 Gold library, domain specificities, and binding affinities of selected scFvs.

ScFv Clones	CDR3 V_H_	CDR3 V_L_	ScFv Library	VEGFR-2 Domain Specificity	Binding Affinity Kd (nM)
G3	P G F S A	D P R G A H	DP47DPL16	Domain 6	137
F1	P G A S A	A P G G A Y	DP47DPL16	Domain 7	6800
A7	GLWGGMDY	QQWNTYPYT	Mouse synthetic V-gene	Domains 2–3	92.6

## Supplementary Materials

Supplementary materials can be found at http://www.mdpi.com/1422-0067/19/5/1334/s1.

## Abbreviations

BSA	Bovine serum albumin
CDR	Complementarity determining region
DARPins	Designed ankyrin repeat proteins
DMEM	Dulbecco’s modified eagle’s medium
ECD	Extracellular domain
*E. coli*	*Escherichia coli*
EGFR	Epidermal growth factor receptor
ELISA	Enzyme linked immunosorbent assay
Fab	Fragment antigen binding
FSEC	Fluorescence size-exclusion chromatography
HEK293	Human embryonic kidney cells 293
HER2	Human epidermal growth factor receptor 2
HRP	Horseradish peroxidase
HUVECs	Human umbilical vein endothelial cells
IgG	Immunoglobulin G
IPTG	Isopropyl β-d-1-thiogalactopyranoside
ITC	Isothermal titration calorimetry
KDR	Kinase insert domain receptor
OD	Optical density
PAE-KDR	Porcine aortic endothelial cells overexpressing VEGFR-2
PBS	Phosphate buffered saline
PCR	Polymerase chain reaction
PLCγ	Phospholipase C gamma
PVDF	Polyvinylidene fluoride
ScFv	Single chain variable fragment
SDS-PAGE	Sodium dodecylsulfate polyacrylamide gel electrophoresis
SEC	Size-exclusion chromatography
VEGF	Vascular endothelial growth factor
VEGFR	VEGF receptor
V_H_/V_L_	Antibody heavy/light chain variable region
