# Supplementary material for: ScFvs as Allosteric Inhibitors of VEGFR-2: Novel Tools to Harness VEGF Signaling
Source: Int J Mol Sci. 2018 May 1;19(5):1334. doi: 10.3390/ijms19051334 (PMC5983656; doi:10.3390/ijms19051334)
Supplement: Supplementary file 1 [file ijms-19-01334-s001.pdf]

International Journal of Molecular Sciences

MDPI

Supplementary Figures

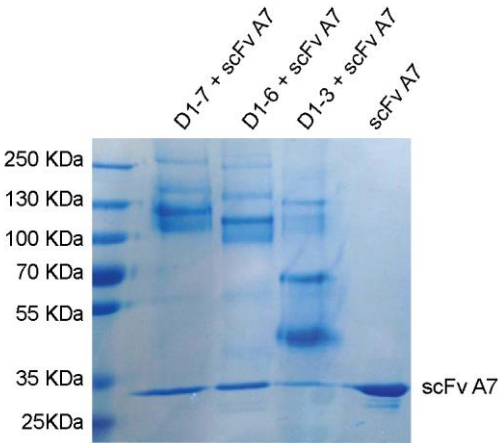

Supplementary Figure 1. Determining domain specificity of scFv A7 by size-exclusion chromatography (SEC). ScFv A7 was incubated with different length constructs of VEGFR-2 ECDs and formed complex were purified with Superdex S-200 chromatography. Proteins from collected peak fractions were concentrated and resolved on 12% SDS-PAGE gel.

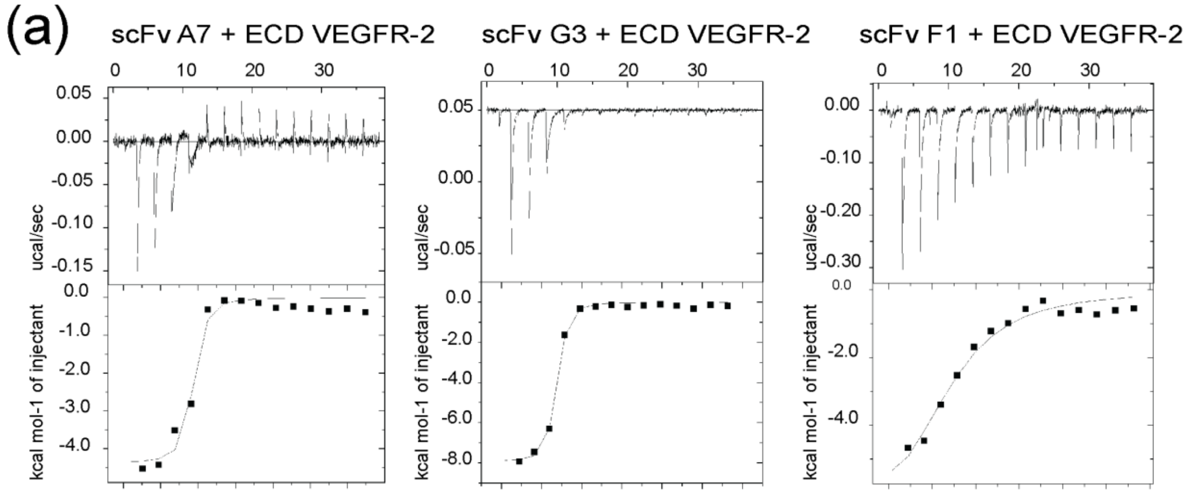

(b)

Complex	Stoichiometric Coefficient, N	Kd (nM)	ΔH (kcal mol^{-1})	TΔS (kcal mol^{-1})
ECD + scFv A7	0.839	92.6	-4383	17
ECD + scFv G3	1.21	137	-7936	3.85
ECD + scFv F1	1.38	6800	-7283	-1.63

Supplementary Figure 2. Thermodynamic analysis of antibody-VEGFR-2 interaction. (a) ITC analysis of scFvs in complex with VEGFR-2 ECD. Raw data and binding isotherms are shown. (b) Calculated thermodynamic parameters of interactions are summarized.

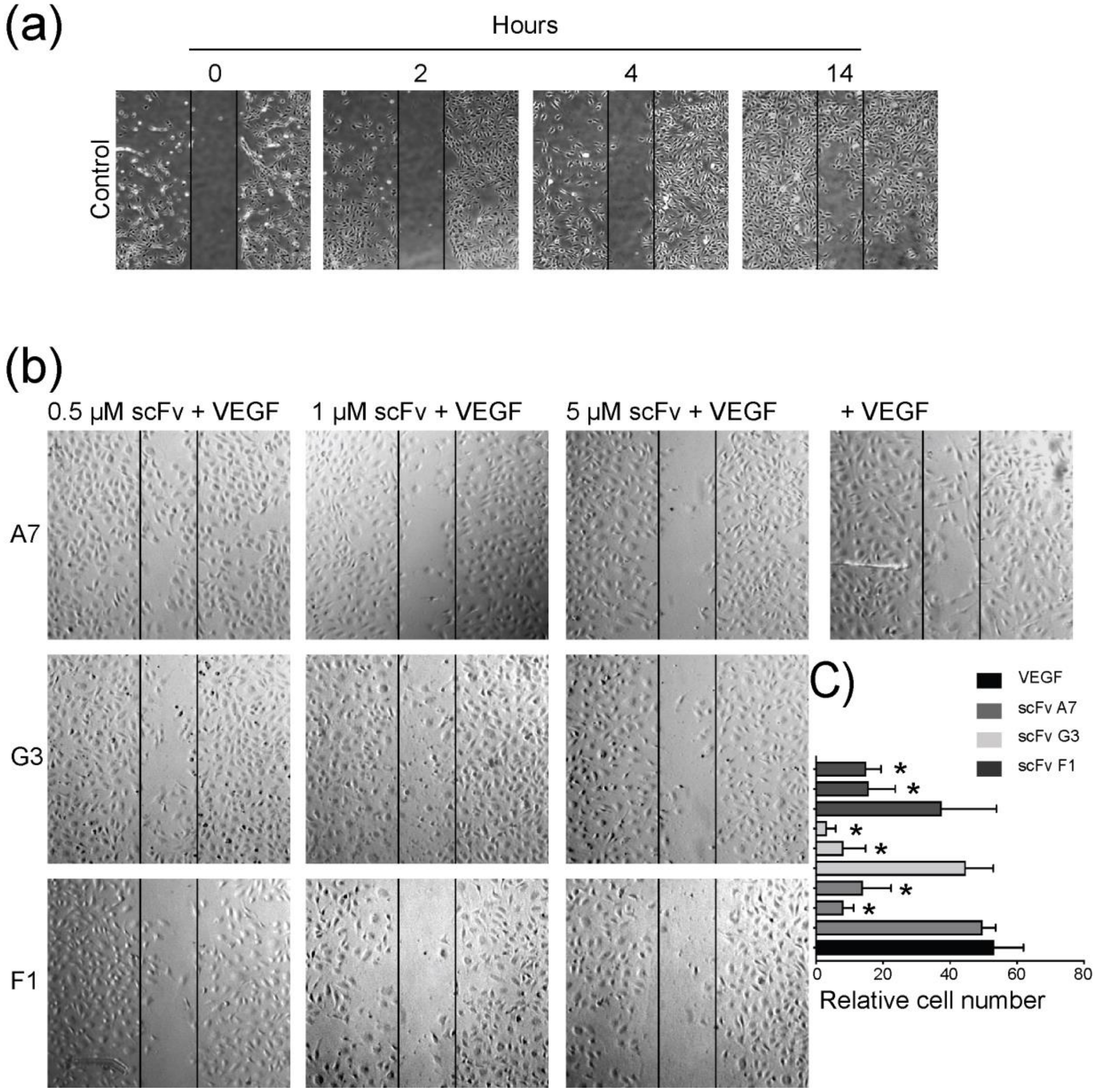

Supplementary Figure 3. ScFvs inhibit cell migration of HUVE cells (a) Time course of VEGF-induced cell migration. (b) Huve cells exposed to increasing concentrations of scFvs. (c) Quantification of data shows the comparison of the relative number of migrating cells in the presence and absence of scFv antibodies. Presented results are mean of three independent experiments where error bars represent ± Standard Deviation (SD). The statistical significance was investigated with ordinary 1-way ANOVA using Dunnett's test and indicated by * representing $P<0.05$.
